# Total temporomandibular joint replacement in recurrent temporomandibular joint ankylosis: a case report

**DOI:** 10.1093/jscr/rjad426

**Published:** 2023-07-20

**Authors:** Dilip Rauniyar, Chandan Upadhyaya, Nitesh Chaurasia, Mamata Shakya, Siddhartha Sharma

**Affiliations:** Department of Oral and Maxillofacial Surgery, KUSMS, Dhulikhel, Nepal; Department of Oral and Maxillofacial Surgery, KUSMS, Dhulikhel, Nepal; Department of Oral and Maxillofacial Surgery, KUSMS, Dhulikhel, Nepal; Department of Oral and Maxillofacial Surgery, KUSMS, Dhulikhel, Nepal; Department of Oral and Maxillofacial Surgery, KUSMS, Dhulikhel, Nepal

## Abstract

Total temporomandibular joint replacement (TMJR) is a surgical procedure in which end-stage temporomandibular joint disorders are replaced with an alloplastic prosthesis between the mandible and the base of the skull when autogenous grafts are inadvisable. These alloplastic prostheses may be available as stock or custom-made prostheses consisting of the mandibular condyle and glenoid fossa components. Although the first total temporomandibular joint prosthesis was used in the 1960s, we present the case of a 20-year-old female patient, probably the first case of total temporomandibular joint arthroplasty in Nepal, performed at Dhulikhel Hospital in Kavrepalanchok. The patient underwent bilateral TMJR with a custom joint prosthesis for recurrent TMJ ankylosis. Postoperatively, the patient noticed significant improvements in mouth opening, chewing ability, facial esthetics and, most importantly, her self-esteem and confidence.

## INTRODUCTION

Temporomandibular joint ankylosis (TMJA) refers to a fused joint caused by the growth of bone mass that has taken the place of the natural architecture [1]. TMJA can cause issues with mastication, speech, esthetics and airway as obstructive sleep apnea, as well as little to no mouth opening, leading to changes in their physical, psychological, social and quality of life [2–4]. The primary etiology of TMJA has been identified as trauma [2]. The other causes of TMJA include middle ear infections and cancrum oris. A gene for progressive ankylosis that regulates osteoblast differentiation has been identified in recent studies [4, 5]. The management of TMJA ranges from simple osteoarthrectomy to complex reconstruction procedures [6].

For consistent treatment outcomes, several TMJ diseases and abnormalities necessitate restoration with a full joint prosthesis. Having had two prior TMJ surgeries, having connective tissue or autoimmune diseases, the absence of TMJ structures caused by pathology, trauma or congenital deformity, and tumors involving the condyle and mandibular ramus are some of these conditions [7].

Perpetual failure rates with re-ankylosis and the inability to sustain long-term mouth opening have been a concern in TMJA therapy [2]. The problem in many developing countries is a lack of adequate access to healthcare or a lack of surgical expertise in underdeveloped areas; illiteracy, which prevents patients from seeking treatment when ankylosis develops, or fear of pain, which leads to a prolonged nonfunctional joint, which can lead to ankylosis. According to published data, people seek therapy for ankylosis anywhere from immediately to several years later [2]. This is significant because ankylosis, if it develops during childhood, causes severe mandibular development retardation [2]. Management of TMJA with temporomandibular joint replacement (TMJR) can be done using stock or custom joints. The use of TMJ prostheses, when compared with other reconstructive procedures, provides immediate function, reducing the duration of surgery, morbidity (does not require a donor area) and hospitalization time. Custom joints can adapt easily and are made for the available bone and its contours [8]. A stock joint requires bone to be prepared for adaptation of the joint [8]. Though the first use of alloplastic materials as TMJ prostheses for the treatment of severe TMJ pathologies began in the 1960s [7], we present the case of a 20-year-old female patient, likely the first case of TMJR done in Nepal in 2022 at Dhulikhel Hospital, Kavrepalanchok. The patient received bilateral TMJR with a custom-made joint prosthesis for recurring TMJA.

## CASE REPORT

**Figure 1:**
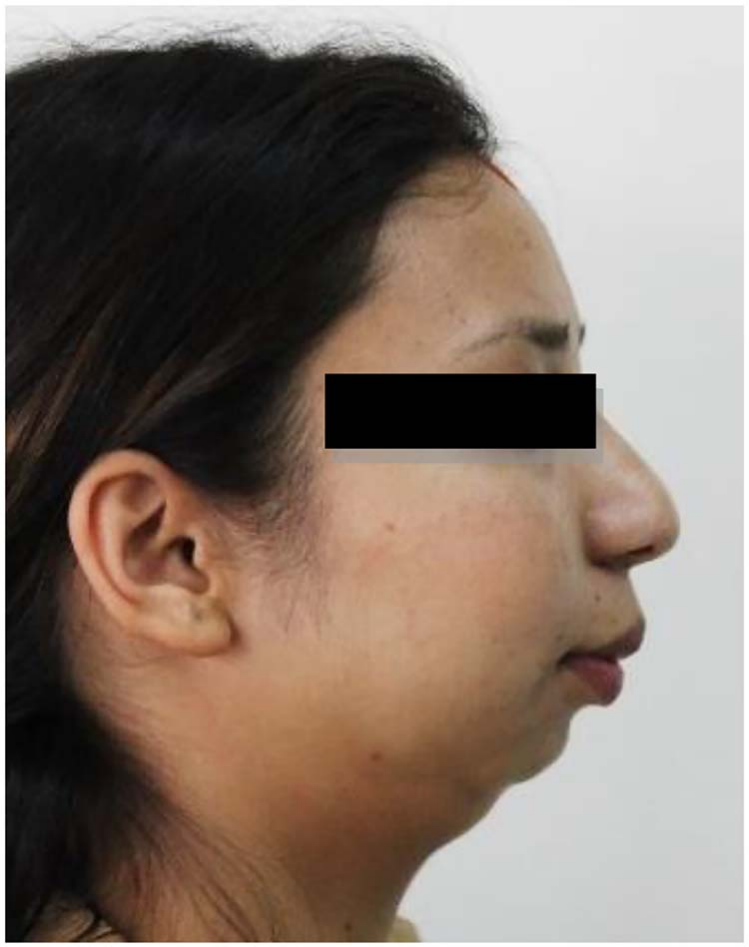
Preoperative clinical images: receding chin with hypoplastic mandible with bird face deformity.

**Figure 2:**
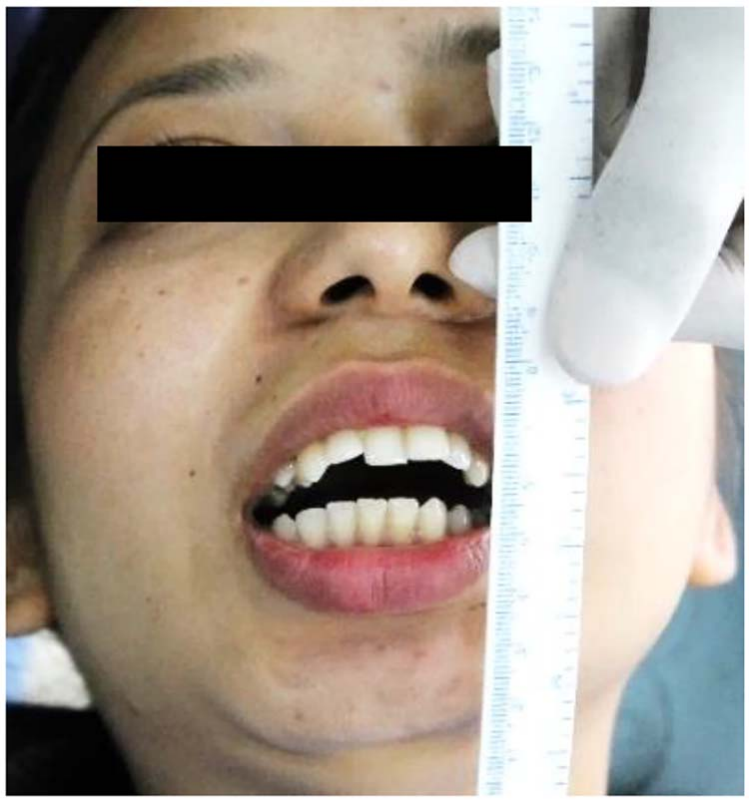
Preoperative clinical images: maximum inter-incisal opening.

A 20-year-old woman was referred to our Department of Oral and Maxillofacial Surgery at Dhulikhel Hospital from another center, complaining mainly of difficulty opening her mouth, difficulty chewing and an unaesthetic appearance (Fig. 1). The patient stated that she had suffered a fall injury 19 years ago in which she had hit her chin area, resulting in a gradual decrease in mouth opening (Fig. 2), so she had undergone surgery to open her mouth at another center at the age of 9 (documentation not available). After surgery, mouth opening increased for a few months, but thereafter the patient noted a decrease in mouth opening and other functional limitations related to jaw movement.

**Figure 3:**
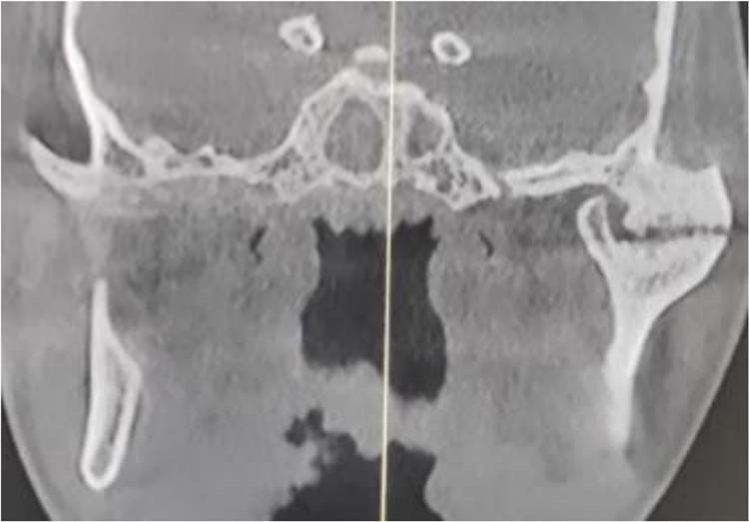
Preoperative computed tomography (coronal section): left-sided TMJA with radiographic suggestion of previous condylectomy at right-sided temporomandibular joint.

The clinical examination of the patient revealed a bird face deformity, chin deviated to the right side, restricted mouth opening of about 5 mm (Fig. 2), class II malocclusion on the right side and class I malocclusion on the left side with protruding upper anterior teeth and an open anterior bite. Cone-beam computed tomography images showed left-sided bony ankylosis of the temporomandibular joint (Fig. 3). Since the patient is skeletally mature, mandibular growth is ceased, and there is recurrent TMJA, reconstruction with an artificial prosthesis can be used as an alternative option [9, 10]. In this case, resection of the left-sided ankylotic bony mass with reshaping of the remaining bilateral ramus and temporal bone was performed for fitting and fixation of a custom made temporomandibular joint prosthesis of Lyka Smith (Fig. 7), Williams Landing, Australia, followed by advancement genioplasty (Figs 8 and 9) [10].

## SURGICAL PROCEDURE

The surgical procedure was performed under general anesthesia (fiber-optic nasal intubation) and consists of two phases: removal of the ankylotic mass and insertion of the temporomandibular joint prosthesis [10]. Alkayat-Bramley and submandibular approaches (Fig. 5) were made bilaterally to gain access to the temporomandibular joint area and the mandibular ramus, respectively. After accessing the temporomandibular joint area, resection of the ankylotic mass and creation of space for the placement of the temporomandibular joint prosthesis were performed bilaterally using cutting guides (Fig. 6) along with a left-sided coronoidectomy. Then, intermaxillary fixation (IMF) was applied. In the initial phase, dummy prostheses were inserted bilaterally to check fit and function, which were eventually replaced by the final custom-made prosthesis. The IMF was then removed, and the patient’s mandible was manipulated to ensure that joint motion was not impeded and that the two prosthetic components fit together properly. Surgical drains were placed bilaterally, and then the surgical sites were closed in layers.

**Figure 4:**
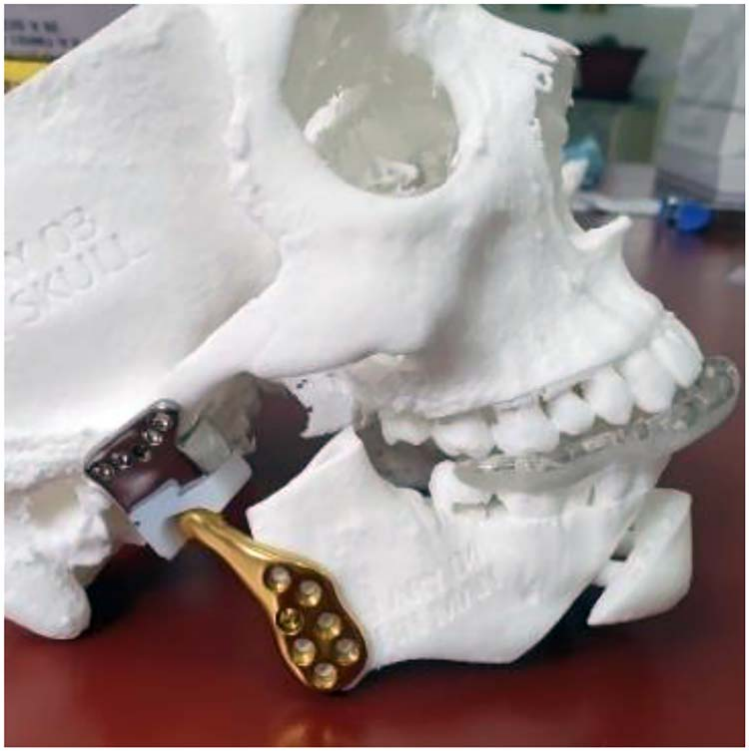
Total temporomandibular joint prosthesis with customized design and 3D printing.

**Figure 5:**
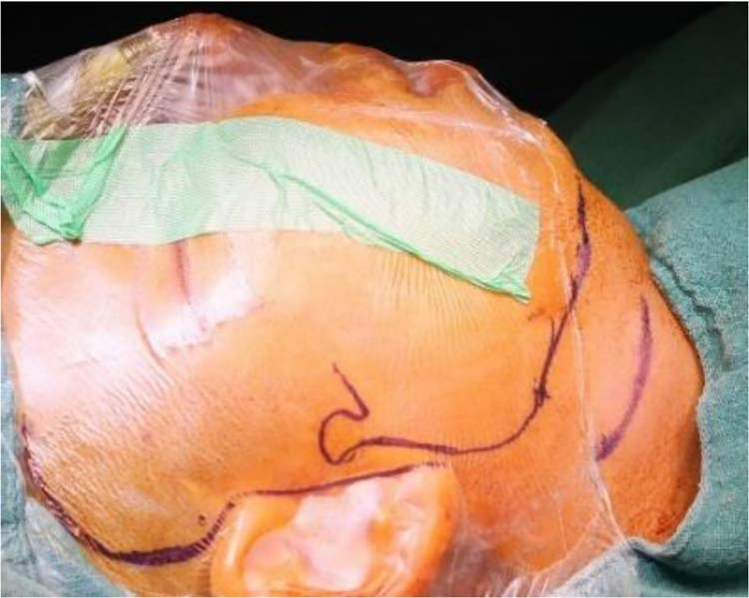
Intraoperative clinical images: marking for Al-Kayat and Bramley approach to the temporomandibular joint.

**Figure 6:**
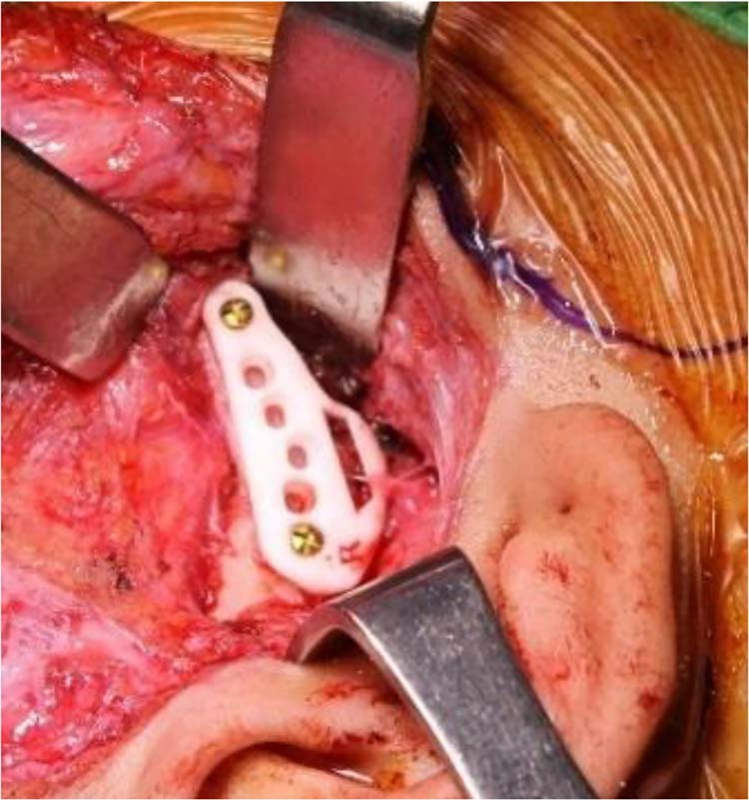
Intraoperative clinical images: placement of patient-specific cutting guides and osteotomy cut for condylectomy.

**Figure 7:**
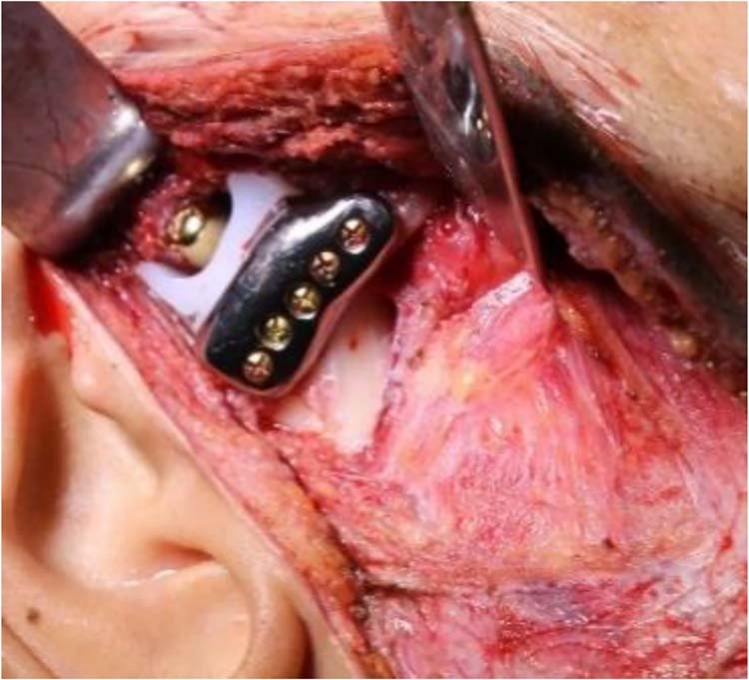
Intraoperative clinical images: positioning and final fixation of the custom prosthesis with screws.

**Figure 8:**
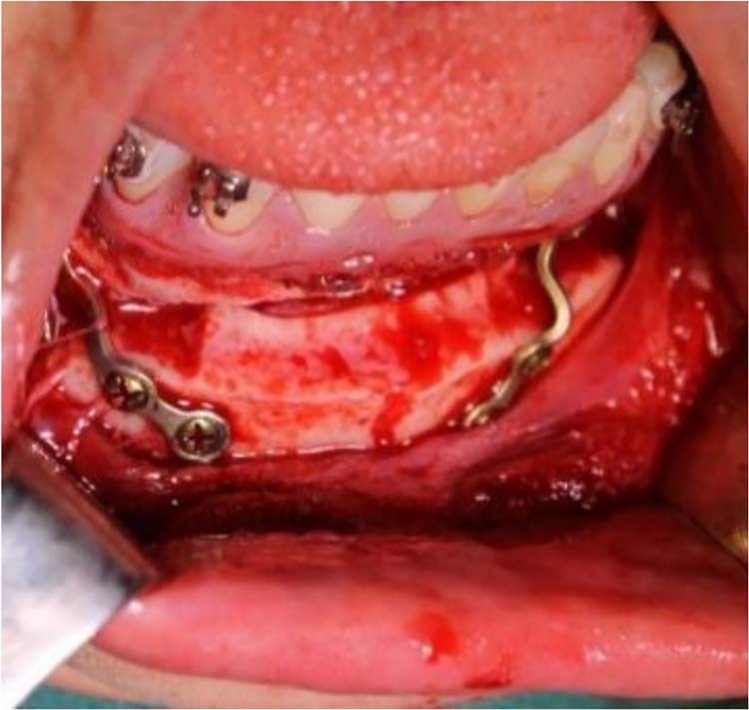
Intraoperative clinical images: horizontal advancement genioplasty and plate fixation.

**Figure 9:**
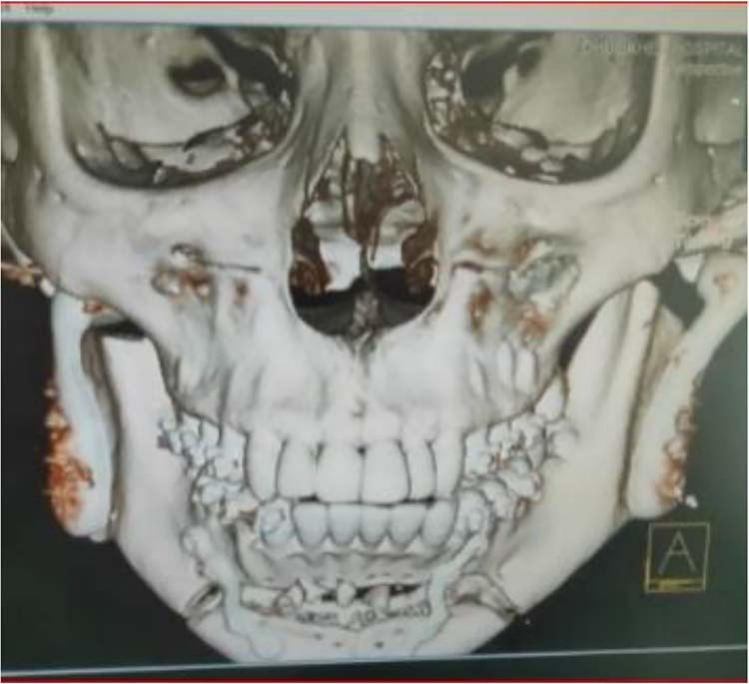
Postoperative 3D reconstruction of the temporomandibular joint prosthesis with horizontal advancement genioplasty.

**Figure 10:**
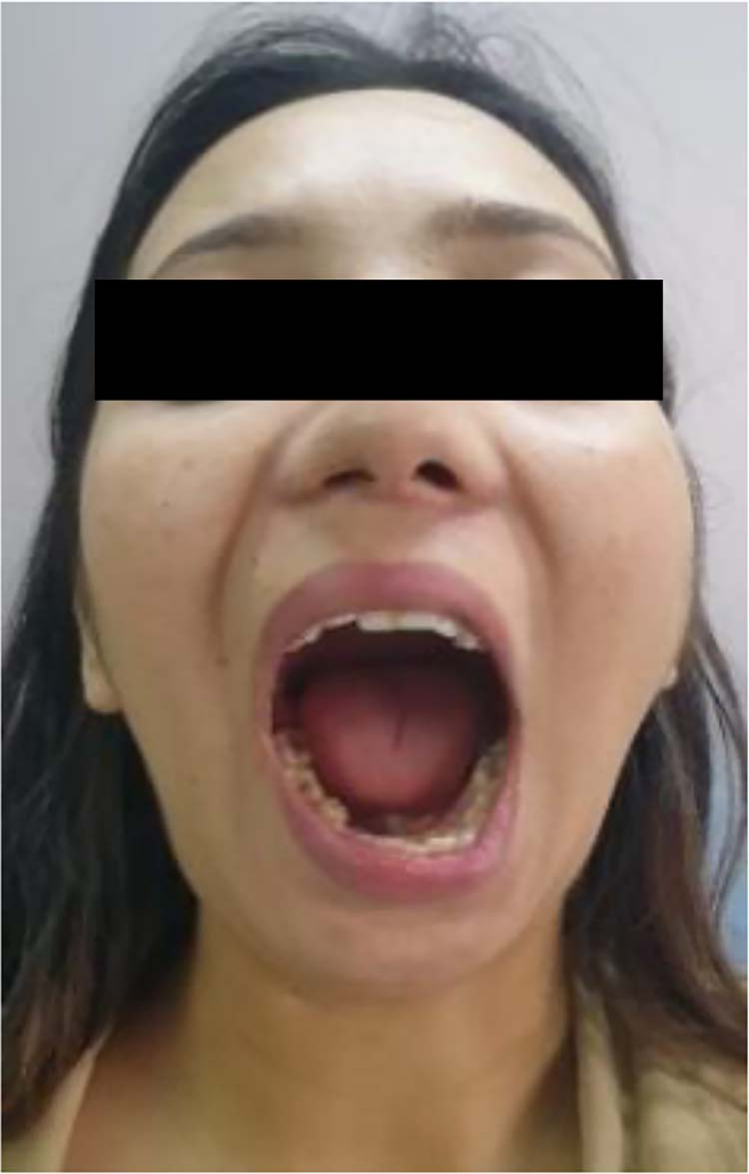
Twelve-month postoperative clinical images and maximum inter-incisal opening.

Postoperatively, passive and active exercises were started 1 week after surgery to rehabilitate the patient’s jaw function [10]. Five follow-up visits were performed (at 1 week, 1, 3, 6 and 12 months). The patient’s mouth opening at the fifth visit was 38 mm (Fig. 10), and she was pain-free on both sides of the temporomandibular joint with the facial nerve intact.

## DISCUSSION

Among the various reconstructive surgical options for the recurrent TMJ ankylosis, to restore the form and function of the joint, the alloplastic joint prosthesis offers a reduction in surgical time without morbidity of the donor site, immediate physical therapy in the postoperative period with improvement of facial symmetry and occlusal stability with a greater predictability of rehabilitation [11]. Alloplastic temporomandibular joint prostheses are available as stock prostheses or as custom-made prostheses. Currently, the use of custom-made prostheses is widely considered by authors, as they confirm that a better distribution of condylar loads on the temporal surface is possible, which increases their longevity, reduces the possibility of fine gap between the host bone and the prosthesis, removes less intact bone for component fitting and reduces perforation of the surrounding anatomical structure [12].

There are several TMJ prosthesis systems on the market. We used the Lyka-Smith TMJ prosthesis system (Fig. 4), which consists of three components: the fossa, the fossa bearing and the ramus components. The fossa-glenoid component was made of ultra-high molecular weight polyethylene, whereas the condylar/mandibular component was made of Co-Cr-Mo alloy with titanium surfaces [10].

Although the first use of alloplastic materials as a TMJ prosthesis to treat severe TMJ pathology began in the 1960s, the case of a 20-year-old female patient at our center, Dhulikhel Hospital, in 2022 was probably the first case of TMJR in Nepal. The patient received bilateral TMJR with a custom joint prosthesis for recurrent TMJ ankylosis [13]. Postoperatively, the patient’s mouth opening was 38 mm (Fig. 10) at 9 months and was pain-free on both sides of the temporomandibular joint with the facial nerve intact, consistent with the success rate described by Mercuri and Wolford *et al.* (1995) [7].

In a 2-year multicenter study of 215 multiply operated TMJ patients (363 joints) reconstructed with the Concepts TMJ prosthesis, it was found to be associated with a 49% reduction in pain, a 43% improvement in jaw function and a 31% increase in maximum jaw opening [7]. Wolford *et al.* (1997) published a 5-year follow-up analysis of 36 patients with 65 TMJs restored with the TMJ Concepts joint prosthesis [7]. The overall success rate for long-term occlusal and skeletal stability after reconstruction was 90%, and pain reduction was noted in 89% of patients. This study led to FDA approval of the TMJ concepts device for use as a total TMJ prosthesis [7].

Regarding the long-term aspects, the lifespan of the TMJ prosthesis and a large amount of follow-up data are not known yet. Nevertheless, alloplastic total TMJ replacement is considered a reliable procedure for recurrent TMJ ankylosis in terms of safety and longevity of the material, based on several recent studies’ results [12].

## References

[1] Andrade NN, Kapoor P, Mathai P, Gupta V, Lakshmi VK, Sharma S. Management of paediatric ankylosis. J Oral Biol Craniofacial Res 2023;13:191–201. 10.1016/j.jobcr.2023.01.006 PMC9860352 36691651

[2] Roychoudhury A, Yadav P, Bhutia O, Mane R, Yadav R, Goswami D, et al. Alloplastic total joint replacement in management of temporomandibular joint ankylosis. J Oral Biol Craniofacial Res 2021;11:457–65. 10.1016/j.jobcr.2021.05.006 PMC8282594 34295642

[3] Elgazzar RF, Abdelhady AI, Saad KA, Elshaal MA, Hussain MM, Abdelal SE, et al. Treatment modalities of TMJ ankylosis: experience in Delta Nile, Egypt. Int J Oral Maxillofac Surg 2010;39:333–42. 20149597 10.1016/j.ijom.2010.01.005

[4] Braimah R, Taiwo A, Ibikunle A, Oladejo T, Adeyemi M, Adejobi F, et al. Clinical experience in managing temporomandibular joint ankylosis: five-year appraisal in a Nigerian subpopulation. J Korean Assoc Oral Maxillofac Surg 2018;44:112–9. 29963492 10.5125/jkaoms.2018.44.3.112 PMC6024065

[5] Pilmane M, Skagers A. Growth factors, genes, bone proteins and apoptosis in the temporomandibular joint (TMJ) of children with ankylosis and during disease recurrence. Stomatol Issued Public Inst Odontol Stud Al 2011;13:96–101. 22071418

[6] Jose A, Nagori SA, Virkhare A, Bhatt K, Bhutia O, Roychoudhury A. Piezoelectric osteoarthrectomy for management of ankylosis of the temporomandibular joint. Br J Oral Maxillofac Surg 2014;52:624–8. 24856926 10.1016/j.bjoms.2014.04.012

[7] Wolford LM, Mehra P. Custom-made total joint prostheses for temporomandibular joint reconstruction. Bayl Univ Med Cent Proc 2000;13:135–8. 10.1080/08998280.2000.11927656 PMC1312294 16389366

[8] Carneiro JT, Pimenta E Souza D, De Assis DSFR, De Moraes PH, Neves Filho FDS. Reconstruction of TMJ with prosthesis joint. In: Motamedi MHK, ed. A Textbook of Advanced Oral and Maxillofacial Surgery. Vol. 3. InTech; 2016.

[9] Sporniak-Tutak K, Janiszewska-Olszowska J, Kowalczyk R. Management of temporomandibular ankylosis – compromise or individualization – a literature review. Med Sci Monit 2011;17:RA111–6. 21525821 10.12659/MSM.881755 PMC3539597

[10] Guarda-Nardini L, Manfredini D, Ferronato G. Total temporomandibular joint replacement: a clinical case with a proposal for post-surgical rehabilitation. J Craniomaxillofac Surg 2008;36:403–9. 18657432 10.1016/j.jcms.2007.11.007

[11] Moreira CVA, Serra AVP, Silva LOR, Fernandes ACF, De Azevedo RA. Total bilateral TMJ reconstruction for pain and dysfunction: case report. Int J Surg Case Rep. 2018;42:138–44. 29245099 10.1016/j.ijscr.2017.11.063 PMC5730393

[12] Lee SH, Ryu DJ, Kim HS, Kim HG, Huh JK. Alloplastic total temporomandibular joint replacement using stock prosthesis: a one-year follow-up report of two cases. J Korean Assoc Oral Maxillofac Surg 2013;39:297. 24516821 10.5125/jkaoms.2013.39.6.297 PMC3912785

[13] Gupta V, Mehrotra D, Malhotra S, Kumar S, Agarwal G, Pal U. An epidemiological study of temporomandibular joint ankylosis. Natl J Maxillofac Surg 2012;3:25. 23251054 10.4103/0975-5950.102146 PMC3513805

